# Inter-assay variability of next-generation sequencing-based gene panels

**DOI:** 10.1186/s12920-022-01230-y

**Published:** 2022-04-15

**Authors:** Pham Nguyen Quy, Keita Fukuyama, Masashi Kanai, Tadayuki Kou, Tomohiro Kondo, Masahiro Yoshioka, Junichi Matsubara, Tomohiro Sakuma, Sachiko Minamiguchi, Shigemi Matsumoto, Manabu Muto

**Affiliations:** 1 Department of Therapeutic Oncology, Graduate School of Medicine, Kyoto University, Kyoto, Japan; 2 Department of Real World Data Research and Development, Graduate School of Medicine, Kyoto University, Kyoto, Japan; 3 Biomedical Department, Mitsui Knowledge Industry Co., Ltd., Tokyo, Japan; 4 Department of Diagnostic Pathology, Graduate School of Medicine, Kyoto University, Kyoto, Japan

**Keywords:** Next-generation sequencing, Comprehensive genomic profiling, Clinical sequencing, Gene panel test, Allele frequency

## Abstract

**Background:**

Tumor heterogeneity has been known to cause inter-assay discordance among next-generation sequencing (NGS) results. However, whether preclinical factors such as sample type, sample quality and analytical features of gene panel can affect the concordance between two different assays remains largely unexplored.

**Methods:**

Replicate sets of DNA samples extracted from formalin-fixed paraffin-embedded tissues (FFPE) (n = 20) and fresh frozen (FF) tissues (n = 10) were herein analyzed using a tumor-only (TO) and paired tumor–normal (TN) gene panel in laboratories certified by the Clinical Laboratory Improvement Amendment. Reported variants from the TO and TN panels were then compared. Furthermore, additional FFPE samples were sequentially sliced from the same FFPE block and submitted to another TN panel assay.

**Results:**

Substantial discordance (71.8%) was observed between the results of the two panels despite using identical DNA samples, with the discordance rate being significantly higher for FFPE samples (*p* < 0.05). Among the 99 variants reported only in the TO panel, 32.3% were consistent with germline variants, which were excluded in the TN panel, while 30.3% had an allele frequency of less than 5%, some of which were highly likely to be artificial calls. The comparison of two independent TN panel assay results from the same FFPE block also showed substantial discordance rate (55.3%).

**Conclusions:**

In the context of clinical settings, our comparative analysis revealed that inter-NGS assay discordance commonly occurred due to sample types and the different analytical features of each panel.

**Supplementary Information:**

The online version contains supplementary material available at 10.1186/s12920-022-01230-y.

## Background

Next-generation sequencing (NGS) gene panels can analyze hundreds of cancer-related genes for somatic alterations in cancer tissue. This novel technology has great potential for providing detailed profiling of variants, which could help improve diagnostic accuracy and treatment selection in patients with cancer [1–3].

Although a variety of gene panels have been introduced into daily clinical practice, the reproducibility of NGS results and the factors that cause discordance between different NGS panels have remained unclear [4–7]. After performing an inter-laboratory comparison to assess the proficiency of gene panel assays in identifying and reporting variants using formalin-fixed, paraffin-embedded (FFPE) tissues from the same FFPE block, Spence et al. reported high concordance (100%) in the identification of exonic variants with an allele frequency (AF) > 15% among all five clinical laboratories despite using different NGS panels [8]. With the increased interest in non-invasive testing, several studies have been conducted to examine concordance between tumor and plasma mutational profiling. Kuderer et al., who compared reported actionable variants between tumor and plasma panels, found a concordance rate of only 22% [9], while Jovelet et al. reported that 55% of patients enrolled in the Molecular Screening for Cancer Treatment Optimization trial were concordant for mutations after comparing tumor and plasma panels [10]. These studies raised many possibilities regarding the source of tumor–plasma discordance, such as tumor heterogeneity, differences in the timing of testing, assay limit of detection, and analytical processes. In a recent study utilizing identical plasma samples and orthogonal comparison of four plasma NGS panels, Stetson et al. suggested that technical factors might be a major source of assay discordance [11]. Such differences might affect clinical decision-making regarding patient treatment.

Nonetheless, inter-assay variability of tissue panels has remained largely unexplored. To address this issue, results of two different NGS gene panels [tumor-only (TO) and paired tumor–normal (TN) panels] using identical DNA samples analyzed in different laboratories accredited by the Clinical Laboratory Improvement Amendment (CLIA) were compared in 30 patients. Furthermore, the NGS results were compared using a pair of FFPE samples from the same FFPE block in 20 patients.

## Methods

### NGS-based gene panel assays

The TO panel (OncoPrime™) is a hybrid capture-based NGS gene panel assay with a total capture size of 1.33 Mb. This gene panel covers the entire coding region of 215 genes and can detect the rearrangement of 17 selected genes as previously described [12]. Illumina HiSeq 2500 was used for NGS sequencing in a CLIA-certified laboratory (EA Genomics; Morrisville, NC, United States). The minimum input DNA quantity required for creating libraries was 150 ng per sample, while the median depth of coverage was more than 3000. Variant calling software VarPROWL (r20278) was used for variant calling [13].

The paired TN panel (NCC Oncopanel v4, RUO version) is a hybrid capture-based NGS assay with a total capture size of 1.38 Mb. This gene panel covers the entire coding region of 114 genes and can detect the rearrangement of 12 selected genes as previously described [14]. With paired normal samples (DNA extracted from blood), this assay can determine germline variants. NGS was performed in a CLIA-certified and College of American Pathologist-accredited laboratory (Riken Genesis Co., Ltd; Kanagawa, Japan). Variant calling was done using variant calling software (cisCall and GATK) in Mitsui Knowledge Industry (Tokyo, Japan). Thresholds for mean read depth in the TN panel were set according to tumor cellularity, as defined by pathological examination. The threshold was set at 200 for samples with > 50% cellularity, 250 for samples with 20%-50% cellularity, and 500 for samples with < 20% cellularity [14]. Analytical features of the TO and TN panels are compared in Additional file 1: Table S1.

### Patients and samples

Between April 2015 and March 2017, a total of 143 patients with histopathologically confirmed solid tumors underwent TO panel assay at Kyoto University Hospital (Kyoto, Japan). Among the 50 patients with actionable mutations, 30 who were available for archived tumor-derived DNA samples were selected for this study. Patients were classified into three groups according to the characteristics of tissue samples: Group FF (n = 10), fresh frozen (FF) tissue samples; Group FFPE-H (n = 10), FFPE samples that yielded DNA library concentrations ≥ 5 nM; and Group FFPE-L (n = 10), FFPE samples that yielded DNA library concentrations < 5 nM.

DNA extracted from FF tumor tissues (Group FF) or FFPE samples with tumor content ≥ 20% (Group FFPE-H and FFPE-L) were utilized for TO panel assay. Residual DNA samples were stored at -80℃ in the Cancer BioBank of Kyoto University Hospital until TN panel assay analysis.

**Fig. 1:**
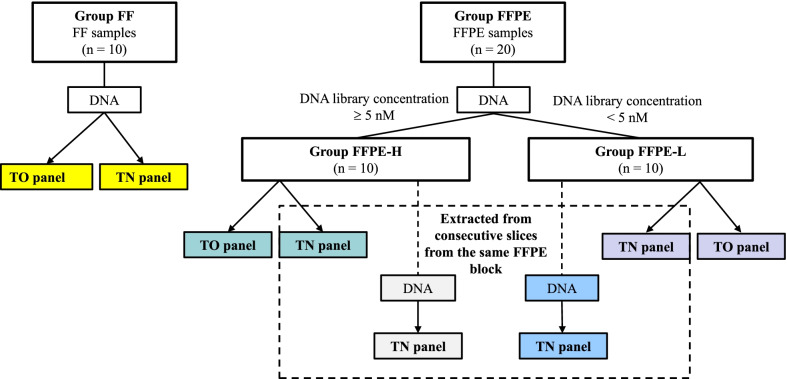
Study design. A total of 30 samples were classified into three groups according to sample type and DNA library concentration: Group FF (n = 10, yellow), fresh frozen (FF) tissue samples; Group FFPE-H (n = 10, green), FFPE samples with DNA library concentrations ≥ 5 nM; and Group FFPE-L (n = 10, purple), FFPE samples with DNA library concentrations < 5 nM. Identical DNA samples were analyzed using tumor-only (TO) and tumor–normal (TN) panel assays. Additional FFPE samples were sliced from the same FFPE block (Group FFPE-H and FFPE-L, n = 20) and submitted for TN panel assay

To investigate the discordance due to different regions of the same tumor, additional FFPE samples were sequentially sliced from the same FFPE block (Group FFPE-H and FFPE-L, n = 20) and submitted to another TN panel assay (Fig. 1).

### Assessment of tumor DNA library concentration

The tumor DNA library was quantified using KAPA Library Quantification Kits (PN KK4824) following the manufacturer’s instructions. In brief, real-time polymerase chain reaction (PCR) was performed to determine the concentration of the 1:1600 dilution of the library in relation to the concentrations of DNA standards. Next, the difference in size between the average fragment length of the library and the DNA standard was estimated using the Agilent TapeStation system (Santa Clara, CA, United States). Finally, the concentration of the undiluted DNA library was calculated by considering the relevant dilution factor [13].

### *Q*-value assessment

Extracted DNA was quantified using a Qubit dsDNA BR Assay Kit (Thermo Fisher Scientific, Waltham, MA, USA) and Qubit 3.0 Fluorometer (Thermo Fisher Scientific). Quantitative PCR analysis of the RPPH1 (RNase P) locus was performed as control, while the ratio of PCR-amplifiable DNA to total double-stranded DNA was used to calculate *Q*-value.

### Definition of target variants for analysis

We verified the detection status of two panels with base substitutions up to a maximum length of 5 bases and InDel were defined as short variants.

### Definition of actionability

In this study, we defined a genomic alteration as actionable if the identified alterations met any of several criteria as previously reported [12]:

1. It can be directly targeted by a US Food and Drug Administration (FDA)-approved drug.
2. It is a signaling pathway component that can be targeted by an FDA-approved drug.
3. It predicts treatment response to an FDA-approved drug.
4. It can be targeted directly or indirectly by an investigational agent that is available in clinical trials.
5. It is a biomarker for which only preclinical data is available.

### Comparative tumor variant analyses

A discordant variant or alteration was defined as that detected only in either of two panels. The concordance rate was calculated by dividing the number of variants found in both panels by the number of variants found in both panels plus all discordant variants. Discordance rate was 100% minus concordance rate.

### Statistical analysis

Difference in concordance rates between the three groups, FF, FFPE-H, and FFPE-L was examined using Fisher’s exact test. Differences in AF between variants detected only in the TO panel and those detected in both panels were evaluated using the Wilcoxon signed-rank test. Correlations were examined using the Spearman correlation method. All reported *p* values used herein were two-sided, with a *p* value < 0.05 indicating statistical significance. All data were analyzed using R (version 3.6.3) and Rstudio (version 1.3.959).

## Results

### Patient characteristics

Clinical and preanalytical characteristics of all 30 patients are summarized in Table 1. The median age was 61.5 years (range, 45–82 years), while 14 patients (46.7%) were males. The most common tumor types tested were pancreatic cancer (n = 7, 23.3%), followed by biliary tract cancers (n = 4, 13.3%), colorectal cancer (n = 4, 13.3%), and cancer of unknown origin (n = 4, 13.3%). Most of the samples were archived within 2 years before the date of sequencing (n = 29; 96.7%).

**Table 1:** Clinical and preanalytical characteristics of 30 patients

Group	No	Age	Sex	Cancer type	Histology	Stage	Concentration of library DNA (nM)	*Q*-value
FF	1	45	M	Pancreas	Adenocarcinoma	IV	8.2	0.85
	2	57	M	Colorectal	Adenocarcinoma	IV	16.2	0.82
	3	56	F	CUP	Adenocarcinoma	IV	18.0	1.00
	4	52	M	Biliary tract	Adenocarcinoma	IV	14.4	0.60
	5	56	M	Pancreas	Adenocarcinoma	IV	13.0	0.79
	6	72	M	Colorectal	Adenocarcinoma	IV	18.5	0.72
	7	82	M	Pancreas	Adenocarcinoma	IV	5.8	0.85
	8	55	F	Esophagus	Squamous cell carcinoma	IV	30.7	1.43
	9	61	F	Pancreas	Neuroendocrine tumor	IV	21.9	1.23
	10	58	F	Biliary tract	Adenocarcinoma	IV	25.1	0.89
FFPE-H	11	47	F	Pancreas	Adenocarcinoma	IV	13.2	1.32
	12	62	F	CUP	Adenocarcinoma	IV	28.5	0.29
	13	72	F	CUP	Squamous cell carcinoma	IV	36.9	0.20
	14	69	M	CUP	Adenocarcinoma	IV	12.1	0.36
	15	64	F	NSCLC	Adenocarcinoma	IV	12.8	0.23
	16	65	F	Gastric	Adenocarcinoma	IV	7.4	1.06
	17	73	M	NSCLC	Adenocarcinoma	IV	4.9	0.43
	18	57	F	Pancreas	Adenocarcinoma	IV	8.1	0.26
	19	73	M	Esophagus	Squamous cell carcinoma	IV	8.2	0.36
	20	69	F	Sarcoma	Leiomyosarcoma	IV	6.1	0.44
FFPE-L	21	36	F	NSCLC	Adenocarcinoma	IV	3.4	0.22
	22	39	F	Colorectal	Adenocarcinoma	IV	1.0	0.21
	23	64	F	Breast	Adenocarcinoma	IV	1.0	0.44
	24	57	M	Esophagus	Squamous cell carcinoma	IV	1.6	0.14
	25	52	F	Biliary tract	Adenocarcinoma	IV	0.2	0.11
	26	61	F	Pancreas	Adenocarcinoma	IV	2.3	0.29
	27	68	M	Biliary tract	Adenocarcinoma	IV	4.2	0.57
	28	66	M	Colorectal	Adenocarcinoma	IV	1.4	0.07
	29	65	M	Gastric	Adenocarcinoma	IV	1.8	0.34
	30	73	M	Bladder	Urothelial carcinoma	IV	4.0	0.27

*M* male, *F* female, *CUP* cancer of unknown primary, *NSCLC* non-small cell lung cancer

### Comparative analyses of tumor variants

The TO panel reported 216 short variants, 8 fusions, and no amplification (TO panel was not designed to report amplifications), while the TN panel reported 84 short variants, 8 fusions, and 4 amplifications. The TO panel reported 75 actionable short variants (34.3%), while the TN panel reported 58 actionable short variants (69.0%) (Additional file 2: Table S2). Although all reported fusions were discordant between the two panels, four fusions in the TN panel were actionable, whereas all of the detected fusions in the TO panel were variants of unknown significance (VUS) (Table 2 and Additional file 3: Table S3).

**Table 2:** Number of reported alterations in the tumor-only and tumor–normal panels

Type of alteration	TO panel	TN panel	Both panels
Amplification	0	4 (4)	0
Fusion	8 (0)	8 (4)	0
Short variant	216 (75)	84 (58)	51 (44)
On common 92 genes	150 (61)	82 (58)	51 (44)
Outside common 92 genes	66 (14)	2 (0)	0

The number of actionable variants is shown in parenthesis

*TO* tumor-only, *TN* tumor–normal

**Fig. 2:**
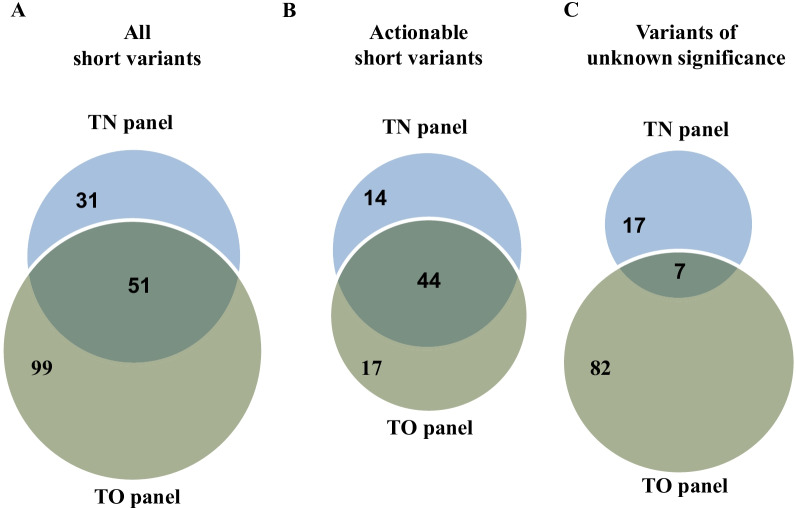
Number of variants reported by the two panels. The number of variants reported in both or each of the tumor-only (TO) and tumor–normal (TN) panels after focusing on 92 genes covered in both panels. Green circles represent variants reported in the TO panel, while blue circles represent variants reported in the TN panel. **A** The number of all short variants reported in each or both of the TO and TN panels. **B** The number of actionable short variants reported in each or both of the TO and TN panels. **C** The number of VUS reported in each or both of the TO and TN panels

Since the two panels target different set of genes, we focused our comparative analysis on short variants in the 92 genes covered in both panels (Additional file 4: Table S4 and Additional file 10: Fig. S1A and B). Among 130 discordant short variants, 99 were detected only in the TO panel, while 31 were found only in the TN panel (Fig. 2A). The number of actionable short variants detected only in the TO panel, only in the TN panel, and in both panels was 17, 14, and 44, respectively. Thus, the concordance rate in identifying actionable variants and VUS between the two panels was 56.7% and 6.6% (Fig. 2B, C).

### Effects of sample types on concordance between NGS results

**Fig. 3:**
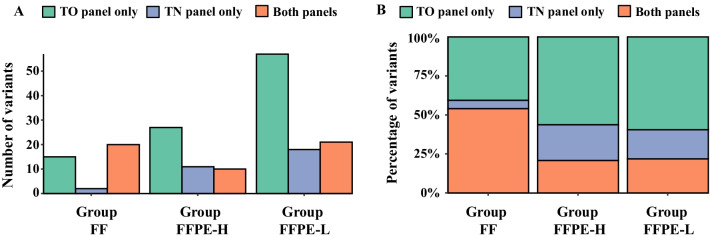
Sample types and concordance rate between the two panels. **A** The number of variants reported in both or each of the tumor-only (TO) and tumor–normal (TN) panels were categorized due to sample type and DNA library concentration (Group FF, FFPE-H, and FFPE-L). The vertical axis shows the number of counted variants. **B** The percentage of reported variants in Group FF, FFPE-H, and FFPE-L were expressed as columns. The vertical axis shows the percentage of variants reported in both or each of the TO and TN panels. The green column represents variants reported only in the TO panel, the blue column represents variants reported only in the TN panel, and the pastel pink column represents variants were reported in both panels

To identify the source of discordance, the effects of sample types on NGS results were analyzed. As shown in Fig. 3A and Additional file 5: Table S5, the number of reported short variants in TO and TN panels increased markedly in Group FFPE-L, with this trend being more prominent in the TO panel. The concordance rate differed significantly between all three groups (Fisher’s exact test, *p* < 0.005), with Group FF (54.1%) showing a higher concordance rate compared to Group FFPE-H (20.4%) and Group FFPE-L (22.1%) (Fig. 3B and Additional file 5: Table S5).

The concordance rate between the two panels was correlated with neither DNA library concentration (r = 0.36, *p* = 0.06) nor *Q*-value (r = 0.18, *p* = 0.36) (Additional file 11: Fig. S2A and 2B).

### AF analysis of discordant variants

**Fig. 4:**
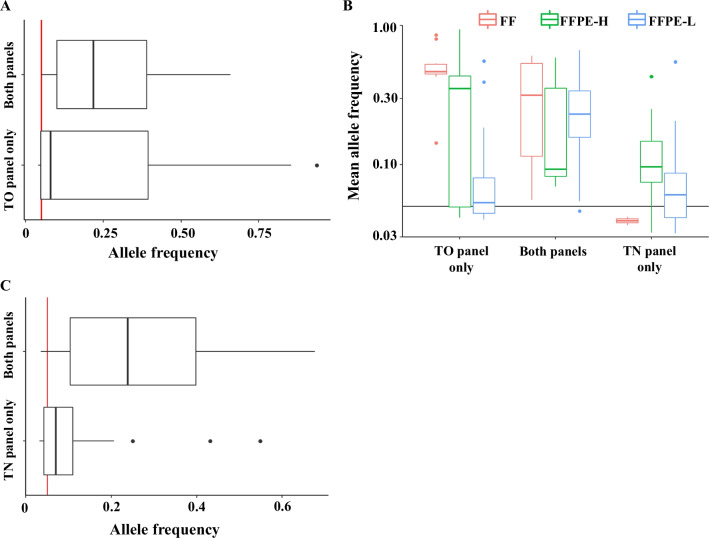
Allele frequency and concordance rate between the two panels. **A** The distribution of allele frequency of variants detected only in the tumor-only (TO) panel and those detected in both panels. **B** The distribution of mean allele frequency of variants reported by the TO panel and/or tumor–normal (TN) panels in Group FF (pastel pink dots), FFPE-H (blue dots), and FFPE-L (green dots). The vertical axis shows the mean allele frequency in logarithmic scale. The horizontal red line and blue line indicate a frequency of 5% and 15%, respectively. **C** The distribution of allele frequency of variants detected only in the TN panel and those detected in both panels

Next, the association between AF and discordant variants were analyzed. As plotted in Fig. 4A, variants detected in the TO panel alone had a lower AF compared to common variants detected in both panels (0.08 and 0.22; Wilcoxon signed-rank test, *p* < 0.005). Further analysis revealed that most of the discordant variants in Group FF and FFPE-H had an AF of approximately 50%, suggesting the possibility of germline variants (Fig. 4B). To support this hypothesis, 32 of the 99 variants (32.3%) reported only in the TO panel were confirmed in normal control bam data of the TN panel (Additional file 6: Table S6).

On the other hand, approximately 30% of the 99 variants had an AF of less than 5%, with such variants being more commonly observed in Group FFPE-L, suggesting the possibility of unreliable calls (Fig. 4B and Additional file 12: S3). Indeed, discordant variants detected with low AF in the TO panel were not detected through the variant calling process of the TN panel (Additional file 12: Fig. S3 and Additional file 6: Table S6).

The median AF of the variants detected only in the TN panel was also lower than that of the concordant variants (0.07 and 0.24; Wilcoxon signed-rank test, *p* < 0.005) (Fig. 4C). Furthermore, 29.5% of these variants had an AF of < 5%, and 66.7% of them belonged to the FFPE-L samples. Approximately 50% of these variants had an AF of < 0.5% in the supportive reads and were derived from the FFPE-L samples.

### Comparison of TN panel assays using different FFPE samples from the same FFPE block

**Fig. 5:**
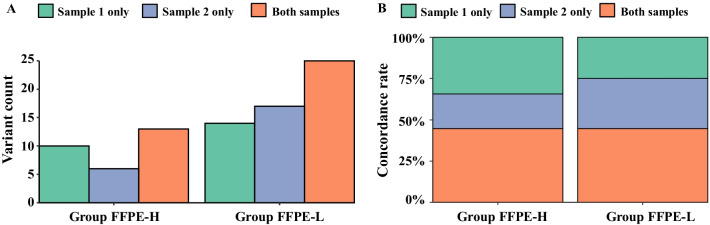
Reported mutations in two different DNA samples. **A** The number of variants reported in both samples or only in one sample were categorized according to Group FFPE-H and FFPE-L. The vertical axis shows the number of counted variants. **B** The percentage of reported variants in Group FFPE-H and FFPE-L were expressed as columns. The vertical axis shows the percentage of variants reported in both samples or in only one sample. The green column represents variants were reported only in initial samples (sample 1), the blue column represents variants reported only in additional samples (sample 2), and the pastel pink column represents variants reported in both samples

To compare the results of independent TN panel assays using different FFPE samples from the same FFPE block, additional FFPE samples were sliced from the same FFPE block (Group FFPE-H and FFPE-L, n = 20) and submitted for another TN panel assay (Fig. 1). Although the TN panel reported a similar number of variants between the first and second assays (n = 62 and 61, respectively), the concordance rate remained as low as 44.7%. As shown in Fig. 5A, B, although the Group FFPE-L had a more reported variants than the Group FFPE-H (n = 56 and 29, respectively), no difference in concordance rate was observed between both groups (44.6% vs. 44.8%; Fisher’s exact test, *p* = 0.58).

## Discussion

To the best of our knowledge, this has been the first study to utilize identical DNA from the same FFPE/FF samples to investigate inter-assay variability between TO and paired TN gene panels, both of which were sequenced and analyzed in CLIA-certified laboratories. Because these panels are commercially available and > 10 institutions have been using them in daily clinical practice in Japan, our comparative analysis is important for understanding the scale and mechanism of discordance between NGS gene panels.

Surprisingly, substantial discordance (71.8%) was observed between the final output from both gene panels despite utilizing identical DNA samples. Considering that FF samples had a significantly higher concordance rate (50.4%) than FFPE samples (21.4%) (Fig. 3B), discordance seems to be partly attributable to DNA quality. Supporting this interpretation, previous studies have reported that DNA degradation is more evident in FFPE compared to FF samples due to formalin-related DNA fragmentation and chemical modification, which potentially increases the incidence of false-positive mutation calls [15–19]. Furthermore, it has been known that FFPE samples do not yield a uniform read-depth coverage across the targeted genes, which can be a probable source of inter-assay discordance [20].

To further investigate the effect of formalin fixation, we analyzed the incidence of C>T/G>A mutations in all samples. As a result, the number of C>T mutations were higher in the samples belonging to Group FFPE-L (Additional file 13: Fig. S4), suggesting the artifact effects of formalin fixation in these samples. This finding might also explain the increased number of short variants in FFPE-L (Fig. 3A). The number of C>T alterations was also higher in the discordant variants, suggesting a relationship between the chemical modification of DNA and discordance between the two NGS panels (Additional file 7: Table S7).

AF analysis of discordant variants showed biased distribution. Indeed, around 30% of discordant variants had an AF of less than 5% (Fig. 4A), most of which were observed in FFPE samples with low DNA library concentration (Group FFPE-L, Fig. 4B and Additional file 12: Fig. S3). Previous studies have reported that variants with low AF may represent subclonal passenger events or non-cancer-derived clones and should therefore be interpreted with caution [21–23]. However, our analysis suggested that the majority of discordant variants with low AF were unreliable calls given that most of them were not detected through the variant calling process of the TN panel and belonged to Group FFPE-L (Additional file 6: Table S6). Further analysis also revealed that the median read depth of the discordant variants reported in the TN panel was significantly lower than that of the concordant variants (189 and 555; Wilcoxon signed-rank test, *p* < 0.005). These findings are consistent with the fact that artificial calls were more common in FFPE samples with low DNA quality as mentioned earlier [15–19].

The difference in analytical pipelines of each NGS gene panel may also contribute to the observed discordance. As shown in Additional file 6: Table S6, among short variants that were found only in the TO panel, all discordant variants observed in FF samples was germline variants which were detected in control bam files of TN panel’s. On the other hand, 96.5% of discordant variants in FFPE-L samples was due to the variant calling process. In FFPE-H group, 55.5% of discordant variants could be attributed to germline variants while 40.7% was not detected by variant caller. Concerning short variants that were found only in the TN panel, discordance observed in FFPE samples was mainly due to variant calling process (66.7% in FFPE-H and 83.3% in FFPE-L).

Although most of the discordant variants were of unknown significance, three clinically relevant variants were reported in the TO panel [*BRCA2* S871* (AF = 0.045, read depth = 2607), *BRCA2* splice site 7977-2A>T (AF = 0.044, read depth = 1684), and *ATM* R1618*(AF = 0.854, read depth = 2884)]. We reviewed the bam files of the TN panel but found no signals related to these *BRCA2* variants. On the other hand, the germline variants of *ATM* were not reported in the TN panel because it was also detected from control samples and was automatically classified as normal variants based on its algorithm.

Another specific distribution was observed in groups FF and FFPE-H such that most of the discordant variants had an AF of approximately 50% (Fig. 4B). Among the 99 genetic variants reported only in the TO panel, 32 (32%) were confirmed in the normal control bam file of the TN panel, suggesting that these variants were of germline origin and were accurately determined by a TN paired panel (Additional file 6: Table S6). In our study, Group FFPE-L had significantly fewer discordances due to heterozygous polymorphisms than Group FFPE-H or FF, which might be due to the fact that, in the TO panel, the maximum number of reportable variants was limited to 14 in their algorithm. Therefore, if the total number of detected variants exceeded 14 (as observed in Group FFPE-L), only the top 14 variants were selected based on their actionability, while other variants were not reported. We consider this the main reason why the discordance rate due to heterozygous polymorphisms was lower in Group FFPE-L. Because matched TN assay is known to increase the accuracy of somatic calls, certain discordant variants might be explained by the difference between the TO and matched TN assays [24–26].

A comparison between independent TN panel assays using different FFPE samples from the same FFPE block also revealed substantial discordance (Fig. 5A, B). Since the tumor cellularity was similar between the samples used in the first and second assays (Additional file 8: Table S8), the discordance appeared to derive from the heterogeneity of the subclones inside each sample or from the low quality of several samples. Several previous studies support the former explanation. The existence of subclones that varied between samples obtained from different regions of the same primary tumor has been demonstrated in breast cancer, renal cell carcinoma, and glioblastoma [27–33]. Moreover, one study showed that intratumor heterogeneity can pose challenges to the tumor genomic profiling of a single tiny tumor sample [6]. In addition to tumor heterogeneity, samples with low DNA quality appeared to affect the high discordant rate. Among the 24 short variants that were reported only in the first assay, 14 were derived from 5 FFPE-L samples, in which 1 sample had 9 discordant short variants. Likewise, among the 23 short variants that were reported only in the second assay, 17 were derived from 8 FFPE-L samples, in which 4 samples had > 3 discordant short variants. Moreover, the incidence of C>T mutations was higher in the samples belonging to Group FFPE-L, suggesting the artifact effects of formalin fixation in these samples (Additional file 9: Table S9).

In our study, all reported fusions were discordant between the two panels. However, it is difficult to speculate the cause of discordance since detection of fusion genes largely depends on target or partner primers of each NGS panel which are not fully disclosed due to trade secrecy.

The current study has several limitations worth noting. First, our sample size (n = 30) was relatively small. Second, there is significant variability in the cancer types, the concentration of library DNA, and the Q-values across the three groups of samples. Third, we could only speculate that discordant short variants with low AF were likely attributed to artificial calls given our inability to confirm it by second orthogonal methods, such as droplet digital PCR. Fourth, we could not extensively control the storage period of samples that might affect the degree of DNA degradation in different samples. Fifth, the current study design could not evaluate the contribution of pre-analytical factors such as library preparation, fragment size selection and nuance parameters related to the NGS assay pipeline.

Finally, considering that our study compared specific TO and TN panels with unique analytical features (Additional file 1: Table S1), our current observations might not be applicable to other panels with different analytical features.

To overcome the limitations of our present study, especially to minimize the confounding factors from clinical settings, future studies involving the enrolment of patients with similar baseline characteristics and application of stricter quality control for the collection, preparation and storage of samples should be considered. In addition, the validation of discordant variants using orthogonal methods, such as Sanger sequencing, will be helpful. In contrast, it is difficult for treating physicians to control the confounding factors emanating from laboratory settings, such as variant callers, minimum AF threshold, and maximum numbers of variants to report, since these parameters are set by independent NGS companies and are not fully disclosed due to trade secrecy.

## Conclusions

In the context of clinical settings, our comparative analysis using identical tumor DNA samples revealed the existence of substantial discordance between the final output of two different gene panels analyzed by CLIA-certified laboratories. The degree of discordance was affected by sample types, DNA quality, and differences in the analytical pipelines of each NGS gene panel. Physicians engaged in daily clinical practice should therefore be aware that discordance is common between independent NGS assays and should pay more attention to the clinical interpretation of variants, especially those with low AF.

## Supplementary Information

**Additional file 1: Table S1.** Analytical features of the two panels.

**Additional file 2: Table S2**. List of all short variants identified by the two panels with all pertinent information.

**Additional file 3: Table S3**. List of all fusions identified by the two panels.

**Additional file 4: Table S4.** List of the 92 genes covered by the two panels.

**Additional file 5: Table S5.** Number of reported short variants in the two panels according to the sample type.

**Additional file 6: Table S6.** Analysis of discordant variants that were detected only in one panel.

**Additional file 7: Table S7**. Number of point mutations in the variants reported in the tumor-only and tumor–normal panels.

**Additional file 8: Table S8.** Tumor cellularity in samples used for the first and second tumor–normal panel assays.

**Additional file 9: Table S9.** Number of point mutations in the concordant and discordant variants reported in the two different tumor–normal panel assays.

**Additional file 10: Figure S1.** The working flow of comparative analysis of tumor variants. (A) The overlapping and different genes between the tumor-only (TO) panel and tumor–normal (TN) panel. (B) Type and number of alterations that were reported and filtered during the comparative analysis.

**Additional file 11: Figure S2.** DNA quality and concordance rate between the two panels. (A) Distribution of reported variants in terms of concordance rate between the two panels (vertical axis) and DNA library concentration (horizontal axis). (B) Distribution of reported variants in terms of concordance rate between the two panels (vertical axis) and *Q*-value (horizontal axis).

**Additional file 12: Figure S3.** Analysis of discordant variants that were reported only in the tumor-only panel. A total of 99 variants detected only in the tumor-only (TO) panel were classified into five categories according to possible causes of discordance and mapped with allele frequency in Group FF, FFPE-H, and FFPE-L.

**Additional file 13: Figure S4.** Incidence of point mutations in short variants reported only in the tumor-only panel. The incidence of C>T/G>A mutations in samples belonging to Group FF, FFPE-H and FFPE-L.

## Data Availability

The datasets generated during the current study are not publicly available because it is possible that individual privacy could be compromised, but are available from the corresponding author on reasonable request.

## Abbreviations

AF: Allele frequency
CLIA: Clinical Laboratory Improvement Amendment
FF: Fresh frozen
FFPE: Formalin-fixed paraffin-embedded
NGS: Next-generation sequencing
PCR: Polymerase chain reaction
TO: Tumor-only
TN: Tumor–normal
