# Supplementary material for: Inter-assay variability of next-generation sequencing-based gene panels
Source: BMC Med Genomics. 2022 Apr 15;15:86. doi: 10.1186/s12920-022-01230-y (PMC9013031; doi:10.1186/s12920-022-01230-y)
Supplement: Supplementary file 1 — Additional file 1: Table S1. Analytical features of the two panels. [file 12920_2022_1230_MOESM1_ESM.docx]

**2 Supplementary Tables**

**Table S1.** Analytical features of the two panels

|  | **TO panel (OncoPrime™)** | **TN panel (NCC oncopanel v4, RUO version)** |
| --- | --- | --- |
| **Sample Type** | Tissue Only | Cancer Tissue and Blood Control |
| **Number of Target Gene** |  |  |
| short variants | 215 | 114 |
| CNV | NA | 114 |
| fusion | 17 | 12 |
| **Strategy** | hybrid capture | hybrid capture |
| **Capture size** | 1.33 Mb | 1.38 Mb |
| **DNA extraction kit** | QIAamp DNA FFPE Tissue kit  (Qiagen, Hilden, Germany) | QIAamp DNA FFPE Tissue kit (Qiagen, Hilden, Germany) and  Maxwell RSC Blood DNA kit (Promega, Fitchburg, WI, USA) |
| **Library prep kit** | SureSelect XT reagent Kit  (Agilent Technologies, Santa Clara, CA, USA) | SureSelect XT reagent (Agilent Technologies, Santa Clara, CA, USA) and KAPA Hyper Prep kit (KAPA Biosystems, Wilmington, MA, USA) |
| **NGS** | Illumina HiSeq 2500 | Illumina MiSeq |
| **Aligmnent program** | BWA and ABRA | BWA |
| **Refference Genome** | hg19 | hg19 |
| **Variant Caller** | VarPROWL | CisCall and GATK |
| **Maximal number of reported variants** | 14 | Unlimited number of variants |
| **Minimal threshold of allele frequency for report** | SNV 4%, Indel 10% | SNV and Indel 3% |
| **Database for annotation** | 1000 Genomes Project, ESP6500, ExAC, OMIM, COSMIC, ClinVar, ClinicalTrials.gov, TCGA, DrugBank, N-of-One disease database, MKI In House database | RefSeq, 1000 Genomes Project, ESP6500, ExAC, HGVD, COSMIC, ClinVar, TCGA, N-of-One disease database, MKI In House database |

TO: Tumor-only, TN: tumor–normal.
