# Supplementary material for: Inter-assay variability of next-generation sequencing-based gene panels
Source: BMC Med Genomics. 2022 Apr 15;15:86. doi: 10.1186/s12920-022-01230-y (PMC9013031; doi:10.1186/s12920-022-01230-y)
Supplement: Supplementary file 2 — Additional file 2: Table S2. List of all short variants identified by the two panels with all pertinent information. [file 12920_2022_1230_MOESM2_ESM.doc]

**Table S2**. List of all short variants identified by the two panels with all pertinent information

| **No.** | **Type** | **Pathogen**  **-icity** | **Position of variant** | **Gene** | **Variant** | **TO panel** | **AF (TO)** | **Dp (TO)** | **TN panel** | **Covered in both panels** | **AF**  **(TN)** | **Dp**  **(TN)** |
| --- | --- | --- | --- | --- | --- | --- | --- | --- | --- | --- | --- | --- |
| **1** | FF | Actionable | chr7:6037058 | PMS2 | splice site 706-4T>- | TRUE | 0.531 | 1108 | FALSE | FALSE |  |  |
| **1** | FF | Actionable | chr12:25398284 | KRAS | G12V | FALSE |  |  | TRUE | TRUE | 0.036570 | 793 |
| **1** | FF | Actionable | chr17:7577082 | TP53 | E286K | TRUE | 0.0545 | 3872 | TRUE | TRUE | 0.064426 | 714 |
| **2** | FF | Actionable | chr17:7579542 | TP53 | D49H | TRUE | 0.798 | 3874 | FALSE | TRUE |  |  |
| **2** | FF | Actionable | chr5:112174920 | APC | H1210fs | TRUE | 0.14 | 5754 | TRUE | TRUE | 0.178147 | 842 |
| **2** | FF | Actionable | chr5:112175466 | APC | S1392* | TRUE | 0.306 | 4254 | TRUE | TRUE | 0.357827 | 626 |
| **2** | FF | Actionable | chr17:7577121 | TP53 | R273C | TRUE | 0.625 | 6524 | TRUE | TRUE | 0.592841 | 894 |
| **3** | FF | VUS | chr13:32914623 | BRCA2 | G2044V | TRUE | 0.45 | 3029 | FALSE | TRUE |  |  |
| **3** | FF | VUS | chr9:21971161 | CDKN2A | H66R | TRUE | 0.536 | 3014 | FALSE | TRUE |  |  |
| **3** | FF | Actionable | chr15:90631838 | IDH2 | R172M | TRUE | 0.194 | 5437 | TRUE | TRUE | 0.186395 | 735 |
| **3** | FF | VUS | chr11:108205775 | ATM | N2697S | TRUE | 0.187 | 3603 | TRUE | TRUE | 0.170153 | 717 |
| **3** | FF | VUS | chr7:128843429 | SMO | T179M | TRUE | 0.457 | 5583 | FALSE | TRUE |  |  |
| **4** | FF | VUS | chr11:108186556 | ATM | L2005V | TRUE | 0.143 | 7868 | FALSE | TRUE |  |  |
| **4** | FF | Actionable | chr3:142274739 | ATR | I774fs | TRUE | 0.0516 | 6937 | FALSE | FALSE |  |  |
| **4** | FF | VUS | chr13:32914277 | BRCA2 | I1929V | TRUE | 0.49 | 10065 | FALSE | TRUE |  |  |
| **5** | FF | Actionable | chr5:112174920 | APC | H1210fs | TRUE | 0.34 | 6922 | TRUE | TRUE | 0.331822 | 1323 |
| **5** | FF | Actionable | chr17:7577120 | TP53 | R273H | TRUE | 0.341 | 7055 | TRUE | TRUE | 0.384256 | 1283 |
| **5** | FF | VUS | chr11:533586 | HRAS | S106L | FALSE |  |  | TRUE | TRUE | 0.041849 | 1601 |
| **6** | FF | VUS | chrX:66765158 | AR | L57Q | TRUE | 0.0527 | 1479 | FALSE | FALSE |  |  |
| **6** | FF | VUS | chr13:32929042 | BRCA2 | A2351G | TRUE | 0.44 | 4516 | FALSE | TRUE |  |  |
| **6** | FF | Actionable | chr19:33792753 | CEBPA | S190P | TRUE | 0.0649 | 1156 | FALSE | FALSE |  |  |
| **6** | FF | VUS | chr16:23619180 | PALB2 | splice site 3350+5G>A | TRUE | 0.427 | 6124 | FALSE | TRUE |  |  |
| **6** | FF | Actionable | chr7:128849189 | SMO | D473N | TRUE | 0.449 | 6274 | FALSE | TRUE |  |  |
| **6** | FF | VUS | chr11:32410710 | WT2 | S483I | TRUE | 0.421 | 6911 | FALSE | FALSE |  |  |
| **7** | FF | Actionable | chr2:178098809 | NFE2L2 | E79V | TRUE | 0.108 | 7063 | TRUE | TRUE | 0.116935 | 1240 |
| **7** | FF | Actionable | chr13:48916767 | RB1 | W99* | TRUE | 0.106 | 4661 | TRUE | TRUE | 0.125191 | 655 |
| **7** | FF | VUS | chr3:47079245 | SETD2 | T2421A | TRUE | 0.529 | 3262 | FALSE | TRUE |  |  |
| **8** | FF | VUS | chr11:108127039 | ATM | Y741C | TRUE | 0.467 | 7520 | FALSE | TRUE |  |  |
| **8** | FF | VUS | chr11:108126976 | ATM | R720H | TRUE | 0.524 | 6950 | FALSE | TRUE |  |  |
| **8** | FF | Actionable | chr22:42525182 | CYP2D6 | F120I | TRUE | 0.812 | 9462 | FALSE | FALSE |  |  |
| **8** | FF | Actionable | chr12:25398285 | KRAS | G12R | TRUE | 0.391 | 7362 | TRUE | TRUE | 0.400815 | 1472 |
| **8** | FF | VUS | chr18:48604778 | SMAD4 | Q534* | TRUE | 0.278 | 2985 | TRUE | TRUE | 0.316265 | 664 |
| **8** | FF | Actionable | chr17:7577610 | TP53 | Splice site 673-2A>G | TRUE | 0.591 | 3344 | TRUE | TRUE | 0.606695 | 1434 |
| **8** | FF | VUS | chr9:37020753 | PAX5 | R31Q | TRUE | 0.293 | 9802 | FALSE | FALSE |  |  |
| **9** | FF | VUS | chr19:33792753 | CEBPA | S190P | TRUE | 0.0438 | 1234 | FALSE | FALSE |  |  |
| **9** | FF | Actionable | chr12:25398284 | KRAS | G12D | TRUE | 0.0506 | 6606 | TRUE | TRUE | 0.060209 | 764 |
| **9** | FF | Actionable | chr18:48573507 | SMAD4 | E31* | TRUE | 0.0642 | 6793 | TRUE | TRUE | 0.058111 | 826 |
| **9** | FF | Actionable | chr17:7578406 | TP53 | R175H | TRUE | 0.0604 | 11413 | TRUE | TRUE | 0.065789 | 988 |
| **9** | FF | VUS | chr19:15276215 | NOTCH3 | A1927T | TRUE | 0.464 | 9931 | FALSE | TRUE |  |  |
| **9** | FF | Actionable | chr14:81609751 | TSHR | R450H | TRUE | 0.5 | 11112 | FALSE | FALSE |  |  |
| **10** | FF | VUS | chr5:112176269 | APC | L1660I | TRUE | 0.481 | 3038 | FALSE | TRUE |  |  |
| **10** | FF | Actionable | chr11:108165729 | ATM | R1618* | TRUE | 0.854 | 2884 | FALSE | TRUE |  |  |
| **10** | FF | Actionable | chr9:21974695 | CDKN2A | Y44* | TRUE | 0.622 | 4416 | TRUE | TRUE | 0.545946 | 555 |
| **10** | FF | Actionable | chr12:25398284 | KRAS | G12V | TRUE | 0.494 | 2612 | TRUE | TRUE | 0.544586 | 628 |
| **10** | FF | Actionable | chr18:48603041 | SMAD4 | Q448* | TRUE | 0.622 | 1847 | TRUE | TRUE | 0.540264 | 683 |
| **10** | FF | Actionable | chr17:7579313 | TP53 | T125R | TRUE | 0.618 | 1125 | TRUE | TRUE | 0.595170 | 704 |
| **10** | FF | VUS | chr14:81534602 | TSHR | V83I | TRUE | 0.392 | 1449 | FALSE | FALSE |  |  |
| **11** | FFPE-H | Actionable | chr13:32893373 | BRCA2 | S76* | TRUE | 0.072 | 2694 | TRUE | TRUE | 0.078652 | 445 |
| **11** | FFPE-H | Actionable | chr8:128750681 | MYC | T73I | TRUE | 0.0937 | 7589 | TRUE | TRUE | 0.079755 | 489 |
| **11** | FFPE-H | Actionable | chr13:49039374 | RB1 | R787* | TRUE | 0.0728 | 6315 | TRUE | TRUE | 0.065263 | 475 |
| **11** | FFPE-H | Actionable | chr17:37868208 | ERBB2 | S310F | FALSE |  |  | TRUE | TRUE | 0.032258 | 589 |
| **11** | FFPE-H | VUS | chrX:48649631 | GATA1 | E39K | TRUE | 0.0689 | 2785 | FALSE | FALSE |  |  |
| **11** | FFPE-H | VUS | chr9:98209213 | PTCH1 | R1442Q | TRUE | 0.481 | 3105 | FALSE | TRUE |  |  |
| **11** | FFPE-H | Actionable | chrX:44918582 | KDM6A | W355* | FALSE |  |  | TRUE | TRUE | 0.078616 | 318 |
| **12** | FFPE-H | VUS | chr2:29462550 | ALK | P784L | FALSE |  |  | TRUE | TRUE | 0.070151 | 727 |
| **12** | FFPE-H | VUS | chr7:6017227 | PMS2 | R813W | TRUE | 0.264 | 1323 | FALSE | FALSE |  |  |
| **12** | FFPE-H | VUS | chr22:41566478 | EP300 | P1452R | TRUE | 0.567 | 7116 | TRUE | TRUE | 0.605230 | 1568 |
| **12** | FFPE-H | VUS | chr7:116411667 | MET | L967P | TRUE | 0.0713 | 3493 | TRUE | TRUE | 0.096096 | 666 |
| **12** | FFPE-H | Actionable | chr3:178936082 | PIK3CA | E542K | TRUE | 0.481 | 4797 | TRUE | TRUE | 0.293754 | 1457 |
| **12** | FFPE-H | VUS | chr12:112891066 | PTPN11 | S134fs | TRUE | 0.201 | 2957 | FALSE | FALSE |  |  |
| **12** | FFPE-H | Actionable | chr17:7579710 | TP53 | N29fs*15 | FALSE |  |  | TRUE | TRUE | 0.431507 | 146 |
| **13** | FFPE-H | VUS | chrX:66765161 | AR | Q58L | TRUE | 0.0885 | 949 | FALSE | FALSE |  |  |
| **13** | FFPE-H | VUS | chr22:29091133 | CHEK2 | A453P | TRUE | 0.425 | 4294 | FALSE | TRUE |  |  |
| **13** | FFPE-H | VUS | chr15:88799324 | NTRK3 | V21F | TRUE | 0.393 | 2826 | FALSE | TRUE |  |  |
| **13** | FFPE-H | Actionable | chr7:6037058 | PMS2 | splice site 706-4T>- | TRUE | 0.381 | 2215 | FALSE | FALSE |  |  |
| **13** | FFPE-H | Actionable | chr19:1207088 | STK11 | S59fs | TRUE | 0.147 | 2397 | FALSE | TRUE |  |  |
| **13** | FFPE-H | Actionable | chr12:25398284 | KRAS | G12D | TRUE | 0.0861 | 4288 | TRUE | TRUE | 0.109504 | 484 |
| **14** | FFPE-H | VUS | chr14:105241276 | AKT1 | T211I | TRUE | 0.0488 | 287 | FALSE | TRUE |  |  |
| **14** | FFPE-H | VUS | chr14:105243104 | AKT1 | C60F | TRUE | 0.0496 | 665 | FALSE | TRUE |  |  |
| **14** | FFPE-H | VUS | chrX:66765280 | AR | Q98fs | TRUE | 0.0496 | 242 | FALSE | FALSE |  |  |
| **14** | FFPE-H | VUS | chr16:68772300 | CDH1 | R50H | TRUE | 0.0532 | 771 | FALSE | FALSE |  |  |
| **14** | FFPE-H | VUS | chr13:28610157 | FLT3 | A445S | TRUE | 0.425 | 4327 | FALSE | TRUE |  |  |
| **14** | FFPE-H | VUS | chr13:28610156 | FLT3 | A445V | TRUE | 0.429 | 4348 | FALSE | TRUE |  |  |
| **14** | FFPE-H | VUS | chr5:180048668 | FLT4 | R632S | TRUE | 0.043 | 1976 | FALSE | TRUE |  |  |
| **14** | FFPE-H | VUS | chr7:116381014 | MET | V546M | TRUE | 0.538 | 5196 | FALSE | TRUE |  |  |
| **14** | FFPE-H | Actionable | chr7:6037058 | PMS2 | splice site 706-4T>- | TRUE | 0.515 | 1616 | FALSE | FALSE |  |  |
| **14** | FFPE-H | VUS | chr9:98231061 | PTCH1 | A741V | TRUE | 0.468 | 4209 | FALSE | TRUE |  |  |
| **14** | FFPE-H | VUS | chr7:128829153 | SMO | V54E | TRUE | 0.0649 | 339 | FALSE | TRUE |  |  |
| **14** | FFPE-H | VUS | chr5:1253878 | TERT | A1122S | TRUE | 0.0458 | 1158 | FALSE | FALSE |  |  |
| **14** | FFPE-H | VUS | chr11:32456443 | WT1 | E150G | TRUE | 0.0447 | 851 | FALSE | FALSE |  |  |
| **15** | FFPE-H | VUS | chr9:133756051 | ABL1 | D560N | TRUE | 0.542 | 3385 | FALSE | TRUE |  |  |
| **15** | FFPE-H | Actionable | chr12:25398281 | KRAS | G13D | TRUE | 0.0938 | 5065 | TRUE | TRUE | 0.069252 | 361 |
| **15** | FFPE-H | Actionable | chr9:21971162 | CDKN2A | H66fs*80 | FALSE |  |  | TRUE | TRUE | 0.088421 | 475 |
| **15** | FFPE-H | Actionable | chr7:6022626 | PMS2 | splice site 706-4T>- | TRUE | 0.42 | 776 | FALSE | FALSE |  |  |
| **15** | FFPE-H | VUS | chr19:10600505 | KEAP1 | W450C | FALSE |  |  | TRUE | FALSE | 0.137026 | 343 |
| **15** | FFPE-H | VUS | chr5:149497181 | PDGFRB | S1046N | FALSE |  |  | TRUE | TRUE | 0.104317 | 278 |
| **15** | FFPE-H | VUS | chr4:106156157 | TET2 | C353F | TRUE | 0.473 | 4713 | FALSE | FALSE |  |  |
| **16** | FFPE-H | VUS | chrX:66765125 | AR | S46N | TRUE | 0.467 | 1907 | FALSE | FALSE |  |  |
| **16** | FFPE-H | Actionable | chr3:142274739 | ATR | I774fs | TRUE | 0.0464 | 7581 | FALSE | FALSE |  |  |
| **16** | FFPE-H | VUS | chr11:69457832 | CCND1 | F78L | TRUE | 0.0437 | 1214 | FALSE | TRUE |  |  |
| **16** | FFPE-H | Actionable | chr17:7578406 | TP53 | R175H | TRUE | 0.359 | 4528 | TRUE | TRUE | 0.327381 | 168 |
| **16** | FFPE-H | VUS | chr7:55087020 | EGFR | A17E | TRUE | 0.0417 | 744 | FALSE | TRUE |  |  |
| **16** | FFPE-H | VUS | chr17:37883959 | ERBB2 | D1144H | TRUE | 0.937 | 25992 | FALSE | TRUE |  |  |
| **16** | FFPE-H | VUS | chr6:160390393 | IGF2R | Q39K | TRUE | 0.056 | 339 | FALSE | FALSE |  |  |
| **16** | FFPE-H | VUS | chr9:98270507 | PTCH1 | D46A | TRUE | 0.0412 | 1312 | FALSE | TRUE |  |  |
| **16** | FFPE-H | VUS | chr7:128850877 | SMO | K575M | TRUE | 0.449 | 6179 | FALSE | TRUE |  |  |
| **16** | FFPE-H | VUS | chr19:1219328 | STK11 | M127R | TRUE | 0.0458 | 2052 | FALSE | TRUE |  |  |
| **16** | FFPE-H | VUS | chr5:1294058 | TERT | R315C | TRUE | 0.0484 | 2064 | FALSE | FALSE |  |  |
| **17** | FFPE-H | VUS | chr15:91298125 | BLM | M348I | TRUE | 0.541 | 9109 | FALSE | FALSE |  |  |
| **17** | FFPE-H | VUS | chr1:97771751 | DPYD | A721T | TRUE | 0.483 | 7558 | FALSE | FALSE |  |  |
| **17** | FFPE-H | VUS | chr4:55156697 | PDGFRA | D1033V | TRUE | 0.393 | 5346 | FALSE | TRUE |  |  |
| **17** | FFPE-H | VUS | chr3:47163843 | SETD2 | M761I | TRUE | 0.351 | 13726 | FALSE | TRUE |  |  |
| **18** | FFPE-H | VUS | chr7:6042242 | PMS2 | A127T | TRUE | 0.474 | 3514 | FALSE | FALSE |  |  |
| **18** | FFPE-H | Actionable | chr12:25398284 | KRAS | G12D | FALSE |  |  | TRUE | TRUE | 0.053968 | 315 |
| **18** | FFPE-H | Actionable | chr17:7577538 | TP53 | R248Q | TRUE | 0.0487 | 2113 | FALSE | TRUE |  |  |
| **19** | FFPE-H | VUS | chrX:48652498 | GATA1 | P390L | TRUE | 0.0653 | 429 | FALSE | FALSE |  |  |
| **19** | FFPE-H | VUS | chr10:8115837 | GATA3 | A396T | TRUE | 0.506 | 1905 | FALSE | FALSE |  |  |
| **19** | FFPE-H | VUS | chr4:153268137 | FBXW7 | R224Q | FALSE |  |  | TRUE | TRUE | 0.250000 | 20 |
| **19** | FFPE-H | VUS | chr9:98209213 | PTCH1 | R1442Q | TRUE | 0.377 | 546 | FALSE | TRUE |  |  |
| **19** | FFPE-H | Actionable | chr17:7576880 | TP53 | P322fs | TRUE | 0.177 | 1721 | FALSE | TRUE |  |  |
| **19** | FFPE-H | VUS | chr14:81575012 | TSHR | S250N | TRUE | 0.383 | 1620 | FALSE | FALSE |  |  |
| **20** | FFPE-H | VUS | chr20:30959968 | ASXL1 | V85A | TRUE | 0.0599 | 5792 | FALSE | FALSE |  |  |
| **20** | FFPE-H | VUS | chr7:140434453 | BRAF | A749S | FALSE |  |  | TRUE | TRUE | 0.158730 | 63 |
| **20** | FFPE-H | VUS | chr9:21971161 | CDKN2A | H66R | TRUE | 0.519 | 937 | FALSE | TRUE |  |  |
| **20** | FFPE-H | VUS | chr12:56487665 | ERBB3 | C533Y | FALSE |  |  | TRUE | TRUE | 0.135135 | 37 |
| **20** | FFPE-H | VUS | chr7:116339280 | MET | A48T | TRUE | 0.116 | 4187 | FALSE | TRUE |  |  |
| **20** | FFPE-H | VUS | chr1:120462032 | NOTCH2 | R1895H | TRUE | 0.236 | 1838 | FALSE | TRUE |  |  |
| **20** | FFPE-H | VUS | chr10:43604487 | RET | L358F | TRUE | 0.0485 | 970 | FALSE | TRUE |  |  |
| **20** | FFPE-H | Actionable | chr17:7578555 | TP53 | splice site 376-1G>A | TRUE | 0.426 | 3029 | TRUE | TRUE | 0.288889 | 45 |
| **20** | FFPE-H | VUS | chr5:1294954 | TERT | V51L | TRUE | 0.0579 | 380 | FALSE | FALSE |  |  |
| **20** | FFPE-H | Actionable | chr17:29550505 | NF1 | Q589* | FALSE |  |  | TRUE | TRUE | 0.096154 | 104 |
| **20** | FFPE-H | VUS | chr12:133209313 | POLE | V2025M | FALSE |  |  | TRUE | FALSE | 0.260870 | 23 |
| **20** | FFPE-H | VUS | chr4:106156895 | TET2 | Q599R | TRUE | 0.361 | 4099 | FALSE | FALSE |  |  |
| **20** | FFPE-H | VUS | chr11:32456528 | WT1 | P122S | TRUE | 0.0481 | 457 | FALSE | FALSE |  |  |
| **21** | FFPE-L | VUS | chr14:105241306 | AKT1 | V201A | TRUE | 0.0492 | 1362 | FALSE | TRUE |  |  |
| **21** | FFPE-L | VUS | chr11:108163375 | ATM | R1489H | TRUE | 0.0407 | 7077 | FALSE | TRUE |  |  |
| **21** | FFPE-L | VUS | chr16:347812 | AXIN1 | G565D | TRUE | 0.0429 | 3171 | FALSE | TRUE |  |  |
| **21** | FFPE-L | VUS | chr17:41276095 | BRCA1 | R7C | TRUE | 0.0604 | 2565 | FALSE | TRUE |  |  |
| **21** | FFPE-L | VUS | chr3:41268756 | CTNNB1 | T332S | TRUE | 0.0411 | 2605 | FALSE | TRUE |  |  |
| **21** | FFPE-L | VUS | chr17:37883555 | ERBB2 | G1056D | TRUE | 0.0408 | 1251 | FALSE | TRUE |  |  |
| **21** | FFPE-L | VUS | chr10:123324027 | FGFR2 | S148I | TRUE | 0.0659 | 1609 | FALSE | TRUE |  |  |
| **21** | FFPE-L | VUS | chr4:1795713 | FGFR3 | A18T | TRUE | 0.0407 | 1942 | FALSE | TRUE |  |  |
| **21** | FFPE-L | VUS | chr13:28611327 | FLT3 | I435K | TRUE | 0.0494 | 2450 | FALSE | TRUE |  |  |
| **21** | FFPE-L | Actionable | chr17:7578190 | TP53 | Y220C | TRUE | 0.255 | 2003 | TRUE | TRUE | 0.263158 | 152 |
| **21** | FFPE-L | Actionable | chr6:160448335 | IGF2R | V255fs | TRUE | 0.0626 | 1374 | FALSE | FALSE |  |  |
| **21** | FFPE-L | VUS | chr7:116380910 | MET | T511M | TRUE | 0.0419 | 2579 | FALSE | TRUE |  |  |
| **21** | FFPE-L | Actionable | chr3:178921331 | PIK3CA | splice site 814-1G>A | TRUE | 0.0412 | 1917 | FALSE | TRUE |  |  |
| **21** | FFPE-L | Actionable | chr4:55597498 | KIT | D716N | FALSE |  |  | TRUE | TRUE | 0.047904 | 167 |
| **21** | FFPE-L | VUS | chr20:36031199 | SRC | G440fs | TRUE | 0.0514 | 1732 | FALSE | FALSE |  |  |
| **22** | FFPE-L | VUS | chr16:68844146 | CDH1 | P245L | TRUE | 0.07 | 3229 | FALSE | FALSE |  |  |
| **22** | FFPE-L | VUS | chr3:41275120 | CTNNB1 | C429Y | TRUE | 0.066 | 3862 | FALSE | TRUE |  |  |
| **22** | FFPE-L | VUS | chr22:41553266 | EP300 | D1119N | TRUE | 0.056 | 3445 | FALSE | TRUE |  |  |
| **22** | FFPE-L | VUS | chr22:41574358 | EP300 | Q2215K | TRUE | 0.137 | 710 | FALSE | TRUE |  |  |
| **22** | FFPE-L | VUS | chr17:37881132 | ERBB2 | D821Y | TRUE | 0.0963 | 903 | FALSE | TRUE |  |  |
| **22** | FFPE-L | Actionable | chr5:112175676 | APC | K1462fs | TRUE | 0.228 | 2742 | TRUE | TRUE | 0.325926 | 135 |
| **22** | FFPE-L | Actionable | chr12:25398284 | KRAS | G12V | TRUE | 0.341 | 7091 | TRUE | TRUE | 0.421359 | 515 |
| **22** | FFPE-L | VUS | chr18:48604770 | SMAD4 | R531Q | TRUE | 0.0566 | 2475 | FALSE | TRUE |  |  |
| **22** | FFPE-L | Actionable | chr17:7578259 | TP53 | V197G | TRUE | 0.538 | 883 | TRUE | TRUE | 0.584158 | 101 |
| **22** | FFPE-L | VUS | chr7:128852105 | SMO | R726Q | TRUE | 0.0766 | 1449 | FALSE | TRUE |  |  |
| **22** | FFPE-L | VUS | chr5:1293664 | TERT | R446H | TRUE | 0.129 | 363 | FALSE | FALSE |  |  |
| **22** | FFPE-L | VUS | chr1:11199716 | MTOR | 4873-1G>A | FALSE |  |  | TRUE | TRUE | 0.206030 | 199 |
| **23** | FFPE-L | VUS | chr5:112175408 | APC | P1373S | TRUE | 0.0481 | 2638 | FALSE | TRUE |  |  |
| **23** | FFPE-L | VUS | chr11:108218008 | ATM | G2863S | FALSE |  |  | TRUE | TRUE | 0.042169 | 166 |
| **23** | FFPE-L | VUS | chr2:202149860 | CASP8 | S392L | TRUE | 0.0591 | 4092 | FALSE | FALSE |  |  |
| **23** | FFPE-L | Actionable | chr16:68867305 | CDH1 | S851* | TRUE | 0.0623 | 2390 | FALSE | FALSE |  |  |
| **23** | FFPE-L | VUS | chr22:41572510 | EP300 | R1680H | TRUE | 0.148 | 714 | FALSE | TRUE |  |  |
| **23** | FFPE-L | VUS | chr4:1801512 | FGFR3 | E140K | TRUE | 0.0778 | 2428 | FALSE | TRUE |  |  |
| **23** | FFPE-L | VUS | chr16:9857589 | GRIN2A | W1271* | TRUE | 0.21 | 800 | FALSE | FALSE |  |  |
| **23** | FFPE-L | VUS | chr6:160525908 | IGF2R | S2423L | TRUE | 0.0699 | 1560 | FALSE | FALSE |  |  |
| **23** | FFPE-L | VUS | chr7:116339608 | MET | E157G | TRUE | 0.0499 | 3569 | FALSE | TRUE |  |  |
| **23** | FFPE-L | VUS | chr13:28599052 | FLT3 | H746Y | FALSE |  |  | TRUE | TRUE | 0.031746 | 189 |
| **23** | FFPE-L | VUS | chr4:55143644 | PDGFRA | V626M | TRUE | 0.0926 | 2235 | FALSE | TRUE |  |  |
| **23** | FFPE-L | VUS | chr9:80409404 | GNAQ | Q237R | FALSE |  |  | TRUE | TRUE | 0.073964 | 338 |
| **23** | FFPE-L | VUS | chr15:99500310 | IGF1R | C1248Y | FALSE |  |  | TRUE | TRUE | 0.040936 | 171 |
| **23** | FFPE-L | VUS | chr9:98231290 | PTCH1 | R665C | TRUE | 0.0761 | 4444 | FALSE | TRUE |  |  |
| **23** | FFPE-L | VUS | chr10:43610164 | RET | V706M | TRUE | 0.14 | 1376 | FALSE | TRUE |  |  |
| **23** | FFPE-L | Actionable | chr5:56167756 | MAP3K1 | Q441X | FALSE |  |  | TRUE | TRUE | 0.039062 | 128 |
| **23** | FFPE-L | Actionable | chr7:128850838 | SMO | R562Q | TRUE | 0.116 | 1771 | FALSE | TRUE |  |  |
| **23** | FFPE-L | VUS | chr20:36028594 | SRC | Q312H | TRUE | 0.0565 | 1628 | FALSE | FALSE |  |  |
| **23** | FFPE-L | Actionable | chr17:29550505 | NF1 | Q589X | FALSE |  |  | TRUE | TRUE | 0.039216 | 306 |
| **23** | FFPE-L | VUS | chr19:15276704 | NOTCH3 | A1854V | FALSE |  |  | TRUE | TRUE | 0.059524 | 84 |
| **23** | FFPE-L | VUS | chr19:11170787 | SMARCA4 | G1612D | FALSE |  |  | TRUE | TRUE | 0.066667 | 75 |
| **23** | FFPE-L | Actionable | chr9:135781467 | TSC1 | R500X | FALSE |  |  | TRUE | TRUE | 0.061224 | 98 |
| **23** | FFPE-L | VUS | chr14:81609934 | TSHR | T511M | TRUE | 0.0544 | 1249 | FALSE | FALSE |  |  |
| **24** | FFPE-L | Actionable | chr5:112174700 | APC | D1137fs | FALSE |  |  | TRUE | TRUE | 0.548589 | 319 |
| **24** | FFPE-L | VUS | chr13:32971138 | BRCA2 | P3202L | TRUE | 0.0474 | 3670 | FALSE | TRUE |  |  |
| **24** | FFPE-L | VUS | chr13:32900267 | BRCA2 | T152K | TRUE | 0.0766 | 4244 | FALSE | TRUE |  |  |
| **24** | FFPE-L | VUS | chr10:123274680 | FGFR2 | P413L | TRUE | 0.0476 | 4975 | FALSE | TRUE |  |  |
| **24** | FFPE-L | Actionable | chr5:176523630 | FGFR4 | E641K | TRUE | 0.0771 | 3087 | FALSE | FALSE |  |  |
| **24** | FFPE-L | VUS | chr12:121431395 | HNF1A | R200Q | TRUE | 0.046 | 3739 | FALSE | FALSE |  |  |
| **24** | FFPE-L | VUS | chr6:160483603 | IGF2R | V1208M | TRUE | 0.0788 | 3693 | FALSE | FALSE |  |  |
| **24** | FFPE-L | VUS | chr9:5089689 | JAK2 | V863M | TRUE | 0.0661 | 5099 | FALSE | TRUE |  |  |
| **24** | FFPE-L | VUS | chr7:116381005 | MET | D543N | TRUE | 0.044 | 9028 | FALSE | TRUE |  |  |
| **24** | FFPE-L | Actionable | chr9:98248148 | PTCH1 | R135* | TRUE | 0.0545 | 3909 | FALSE | TRUE |  |  |
| **24** | FFPE-L | VUS | chr5:1264696 | TERT | R889Q | TRUE | 0.0592 | 3596 | FALSE | FALSE |  |  |
| **24** | FFPE-L | VUS | chr4:106157540 | TET2 | R814H | TRUE | 0.0516 | 4748 | FALSE | FALSE |  |  |
| **24** | FFPE-L | Actionable | chr9:135779172 | TSC1 | R692* | TRUE | 0.0753 | 3187 | FALSE | TRUE |  |  |
| **24** | FFPE-L | Actionable | chr13:48951052 | RB1 | splice site 1216-2A>G | TRUE | 0.427 | 2345 | TRUE | TRUE | 0.676966 | 356 |
| **24** | FFPE-L | Actionable | chr17:7578419 | TP53 | E171* | TRUE | 0.663 | 6732 | TRUE | TRUE | 0.664093 | 259 |
| **25** | FFPE-L | VUS | chr5:112176806 | APC | A1839S | TRUE | 0.096 | 3592 | FALSE | TRUE |  |  |
| **25** | FFPE-L | Actionable | chr12:25398284 | KRAS | G12D | TRUE | 0.0662 | 4485 | TRUE | TRUE | 0.042403 | 283 |
| **25** | FFPE-L | VUS | chr16:68845600 | CDH1 | M282I | TRUE | 0.0901 | 2241 | FALSE | FALSE |  |  |
| **25** | FFPE-L | VUS | chr10:123239498 | FGFR2 | S780L | TRUE | 0.184 | 4853 | FALSE | TRUE |  |  |
| **25** | FFPE-L | VUS | chr11:64572600 | MEN1 | G419D | TRUE | 0.114 | 1494 | FALSE | FALSE |  |  |
| **25** | FFPE-L | VUS | chr7:116340051 | MET | K305E | TRUE | 0.0734 | 1022 | FALSE | TRUE |  |  |
| **25** | FFPE-L | VUS | chr7:116435763 | MET | P1303S | TRUE | 0.1 | 6239 | FALSE | TRUE |  |  |
| **25** | FFPE-L | VUS | chr4:55144567 | PDGFRA | D681Y | TRUE | 0.0697 | 3957 | FALSE | TRUE |  |  |
| **25** | FFPE-L | VUS | chr9:80409404 | GNAQ | Q237R | FALSE |  |  | TRUE | TRUE | 0.070175 | 285 |
| **25** | FFPE-L | VUS | chr9:98238392 | PTCH1 | T551M | TRUE | 0.102 | 1920 | FALSE | TRUE |  |  |
| **25** | FFPE-L | Actionable | chr13:49039374 | RB1 | R787* | TRUE | 0.0412 | 7036 | FALSE | TRUE |  |  |
| **25** | FFPE-L | VUS | chr2:198274523 | SF3B1 | R292K | TRUE | 0.0643 | 1120 | FALSE | FALSE |  |  |
| **25** | FFPE-L | VUS | chr2:198265573 | SF3B1 | E862K | TRUE | 0.0652 | 6672 | FALSE | FALSE |  |  |
| **25** | FFPE-L | VUS | chr4:106157246 | TET2 | S716L | TRUE | 0.0678 | 3272 | FALSE | FALSE |  |  |
| **25** | FFPE-L | Actionable | chr17:7578212 | TP53 | R213* | TRUE | 0.133 | 2396 | FALSE | TRUE |  |  |
| **26** | FFPE-L | VUS | chr11:108205756 | ATM | R2691C | TRUE | 0.0523 | 3307 | FALSE | TRUE |  |  |
| **26** | FFPE-L | Actionable | chr1:27100943 | ARID1A | Q1409X | FALSE |  |  | TRUE | TRUE | 0.122222 | 270 |
| **26** | FFPE-L | Actionable | chr13:32937314 | BRCA2 | splice site 7977-2A>T | TRUE | 0.0439 | 1684 | FALSE | TRUE |  |  |
| **26** | FFPE-L | Actionable | chr13:32911104 | BRCA2 | S871* | TRUE | 0.0445 | 2607 | FALSE | TRUE |  |  |
| **26** | FFPE-L | VUS | chr13:32930636 | BRCA2 | V2503I | TRUE | 0.046 | 3869 | FALSE | TRUE |  |  |
| **26** | FFPE-L | Actionable | chr3:178952090 | PIK3CA | G1049R | TRUE | 0.0568 | 3449 | TRUE | TRUE | 0.035250 | 539 |
| **26** | FFPE-L | Actionable | chr17:7578263 | TP53 | R196* | TRUE | 0.0631 | 2013 | TRUE | TRUE | 0.056948 | 439 |
| **26** | FFPE-L | Actionable | chr12:25398284 | KRAS | G12D | FALSE |  |  | TRUE | TRUE | 0.051071 | 607 |
| **26** | FFPE-L | Actionable | chr19:1221319 | STK11 | P281L | TRUE | 0.555 | 3987 | FALSE | TRUE |  |  |
| **27** | FFPE-L | VUS | chr12:52374840 | ACVR1B | R223Q | TRUE | 0.46 | 8674 | FALSE | FALSE |  |  |
| **27** | FFPE-L | VUS | chr19:33792713 | CEBPA | A203V | TRUE | 0.0452 | 310 | FALSE | FALSE |  |  |
| **27** | FFPE-L | Actionable | chr12:25398284 | KRAS | G12D | TRUE | 0.117 | 12556 | TRUE | TRUE | 0.156171 | 794 |
| **27** | FFPE-L | Actionable | chr18:48575102 | SMAD4 | W99* | TRUE | 0.127 | 13125 | TRUE | TRUE | 0.096519 | 632 |
| **27** | FFPE-L | VUS | chr10:43615134 | RET | D850N | TRUE | 0.0458 | 2619 | FALSE | TRUE |  |  |
| **27** | FFPE-L | VUS | chr11:32456528 | WT1 | P122S | TRUE | 0.0499 | 481 | FALSE | FALSE |  |  |
| **28** | FFPE-L | VUS | chr11:108235812 | ATM | L2952F | TRUE | 0.0401 | 3740 | FALSE | TRUE |  |  |
| **28** | FFPE-L | Actionable | chr5:112116592 | APC | R213* | TRUE | 0.158 | 4405 | TRUE | TRUE | 0.203735 | 589 |
| **28** | FFPE-L | Actionable | chr5:112175212 | APC | I1307fs | TRUE | 0.218 | 3007 | TRUE | TRUE | 0.125806 | 310 |
| **28** | FFPE-L | Actionable | chr12:25398284 | KRAS | G12V | TRUE | 0.283 | 4817 | TRUE | TRUE | 0.238193 | 487 |
| **28** | FFPE-L | Actionable | chr3:178936091 | PIK3CA | E545K | TRUE | 0.166 | 5166 | TRUE | TRUE | 0.146470 | 949 |
| **28** | FFPE-L | Actionable | chr17:7577094 | TP53 | R282W | TRUE | 0.387 | 5055 | TRUE | TRUE | 0.291391 | 302 |
| **28** | FFPE-L | VUS | chr2:47637269 | MSH2 | L135F | TRUE | 0.0481 | 5555 | FALSE | TRUE |  |  |
| **28** | FFPE-L | VUS | chr16:23641520 | PALB2 | S652N | TRUE | 0.394 | 12124 | FALSE | TRUE |  |  |
| **28** | FFPE-L | VUS | chr9:98209683 | PTCH1 | Q1285H | TRUE | 0.0428 | 6688 | FALSE | TRUE |  |  |
| **28** | FFPE-L | Actionable | chr10:43607555 | RET | E511K | TRUE | 0.0528 | 3240 | FALSE | TRUE |  |  |
| **28** | FFPE-L | VUS | chr7:128843306 | SMO | R138Q | TRUE | 0.0403 | 11183 | FALSE | TRUE |  |  |
| **29** | FFPE-L | VUS | chr16:68835597 | CDH1 | R63Q | TRUE | 0.0438 | 6137 | FALSE | FALSE |  |  |
| **29** | FFPE-L | VUS | chr22:41553382 | EP300 | M1157I | FALSE |  |  | TRUE | TRUE | 0.090909 | 297 |
| **29** | FFPE-L | Actionable | chr7:55242511 | EGFR | D761N | TRUE | 0.0477 | 6893 | FALSE | TRUE |  |  |
| **29** | FFPE-L | VUS | chr4:1806243 | FGFR3 | R421Q | TRUE | 0.0422 | 5545 | FALSE | TRUE |  |  |
| **29** | FFPE-L | Actionable | chrX:48652236 | GATA1 | Q303* | TRUE | 0.0667 | 3341 | FALSE | FALSE |  |  |
| **29** | FFPE-L | Actionable | chr12:121434361 | HNF1A | G375fs | TRUE | 0.0409 | 5359 | FALSE | FALSE |  |  |
| **29** | FFPE-L | Actionable | chr17:7578442 | TP53 | Y163C | TRUE | 0.215 | 11156 | TRUE | TRUE | 0.234483 | 145 |
| **29** | FFPE-L | VUS | chr19:11132428 | SMARCA4 | E882K | TRUE | 0.0475 | 2630 | FALSE | TRUE |  |  |
| **29** | FFPE-L | VUS | chr17:29528490 | NF1 | R416Q | FALSE |  |  | TRUE | TRUE | 0.037657 | 478 |
| **29** | FFPE-L | VUS | chr14:81422098 | TSHR | S25L | TRUE | 0.0434 | 3870 | FALSE | FALSE |  |  |
| **30** | FFPE-L | VUS | chrX:66905896 | AR | D605N | TRUE | 0.0805 | 1628 | FALSE | FALSE |  |  |
| **30** | FFPE-L | VUS | chr11:108121532 | ATM | R447Q | FALSE |  |  | TRUE | TRUE | 0.115385 | 78 |
| **30** | FFPE-L | VUS | chr6:32166818 | NOTCH4 | R1474* | TRUE | 0.0492 | 1241 | FALSE | FALSE |  |  |
| **30** | FFPE-L | VUS | chr7:6029426 | PMS2 | splice site 1144+5G>C | TRUE | 0.045 | 5443 | FALSE | FALSE |  |  |
| **30** | FFPE-L | VUS | chr11:108165673 | ATM | S1599L | TRUE | 0.203 | 4148 | TRUE | TRUE | 0.256410 | 39 |
| **30** | FFPE-L | VUS | chr11:108218090 | ATM | L2890P | TRUE | 0.255 | 4667 | TRUE | TRUE | 0.396552 | 58 |
| **30** | FFPE-L | Actionable | chr17:7578394 | TP53 | H179R | TRUE | 0.345 | 1517 | TRUE | TRUE | 0.411765 | 17 |
| **30** | FFPE-L | VUS | chr18:48581153 | SMAD4 | P153A | TRUE | 0.212 | 2968 | TRUE | TRUE | 0.179487 | 39 |
| **30** | FFPE-L | VUS | chr10:89711968 | PTEN | H196Y | TRUE | 0.106 | 5369 | FALSE | TRUE |  |  |
