# Supplementary material for: Inter-assay variability of next-generation sequencing-based gene panels
Source: BMC Med Genomics. 2022 Apr 15;15:86. doi: 10.1186/s12920-022-01230-y (PMC9013031; doi:10.1186/s12920-022-01230-y)
Supplement: Supplementary file 3 — Additional file 3: Table S3. List of all fusions identified by the two panels. [file 12920_2022_1230_MOESM3_ESM.docx]

**Table S3.** List of all fusions identified by the two panels

| **Case No.** | **Sample** | **Gene** | **Variant** | **Pathogenicity** | **Concordance** |
| --- | --- | --- | --- | --- | --- |
| 30 | FFPE-L | BCR\|LINGO2 | Fusion | VUS | x(TO only) |
| 27 | FFPE-L | BCR\|PPM1K | Fusion | VUS | x(TO only) |
| 18 | FFPE-H | ETV6\|C3orf67 | Fusion | VUS | x(TO only) |
| 16 | FFPE-H | PDGFRA\|SULF2 | Fusion | VUS | x(TO only) |
| 29 | FFPE-L | RET\|PELI2 | Fusion | VUS | x(TO only) |
| 14 | FFPE-H | ROS1\|EYS | Fusion | VUS | x(TO only) |
| 26 | FFPE-L | TMPRSS2\|DKFZp667J0810 | Fusion | VUS | x(TO only) |
| 20 | FFPE-H | TMPRSS2\|RNA5-8S5 | Fusion | VUS | x(TO only) |
| 20 | FFPE-H | AKT3\|NRG1 | Fusion | Actionable | x(TN only) |
| 19 | FFPE-H | BRAF\|MAGI3 | Fusion | Actionable | x(TN only) |
| 23 | FFPE-L | BRAF\|MAGI3 | Fusion | Actionable | x(TN only) |
| 23 | FFPE-L | CIT\|NRG1 | Fusion | Actionable | x(TN only) |
| 19 | FFPE-H | MAGI3\|PDGFRA | Fusion | VUS | x(TN only) |
| 20 | FFPE-H | NRG1\|CIT | Fusion | VUS | x(TN only) |
| 19 | FFPE-H | PDGFRA\|MAGI3 | Fusion | VUS | x(TN only) |
| 20 | FFPE-H | SLC34A2\|ALK | Fusion | VUS | x(TN only) |
