# Supplementary material for: Inter-assay variability of next-generation sequencing-based gene panels
Source: BMC Med Genomics. 2022 Apr 15;15:86. doi: 10.1186/s12920-022-01230-y (PMC9013031; doi:10.1186/s12920-022-01230-y)
Supplement: Supplementary file 4 — Additional file 4: Table S4. List of the 92 genes covered by the two panels. [file 12920_2022_1230_MOESM4_ESM.docx]

**Table S4.** List of the 92 genes covered by the two panels

| Common 92 genes in both TO and TN panels | | | | | | | | | |  |
| --- | --- | --- | --- | --- | --- | --- | --- | --- | --- | --- |
| 1 | ABL1 | BAP1 | DDR2 | FGFR2 | IGF1R | MAP2K4 | NF1 | PBRM1 | RET | TSC1 |
| 2 | AKT1 | BRAF | EGFR | FGFR3 | IL7R | MAP3K1 | NFE2L2 | PDGFRA | ROS1 | VHL |
| 3 | AKT2 | BRCA1 | EP300 | FGFR4 | JAK1 | MDM2 | NOTCH1 | PDGFRB | SETD2 |  |
| 4 | AKT3 | BRCA2 | ERBB2 | FLT3 | JAK2 | MDM4 | NOTCH2 | PIK3CA | SMAD4 |  |
| 5 | ALK | CCND1 | ERBB3 | GNA11 | JAK3 | MET | NOTCH3 | PIK3R1 | SMARCA4 |  |
| 6 | APC | CDK4 | ERBB4 | GNAQ | KDM6A | MLH1 | NRAS | PIK3R2 | SMARCB1 |  |
| 7 | ARAF | CDKN2A | ESR1 | GNAS | KIT | MSH2 | NTRK1 | PTCH1 | SMO |  |
| 8 | ARID1A | CHEK2 | EZH2 | HRAS | KRAS | MTOR | NTRK2 | PTEN | STAT3 |  |
| 9 | ATM | CREBBP | FBXW7 | IDH1 | MAP2K1 | MYC | NTRK3 | RAF1 | STK11 |  |
| 10 | AXIN1 | CTNNB1 | FGFR1 | IDH2 | MAP2K2 | MYCN | PALB2 | RB1 | TP53 |  |

TO: Tumor-only, TN: tumor–normal.
