# Supplementary material for: Inter-assay variability of next-generation sequencing-based gene panels
Source: BMC Med Genomics. 2022 Apr 15;15:86. doi: 10.1186/s12920-022-01230-y (PMC9013031; doi:10.1186/s12920-022-01230-y)
Supplement: Supplementary file 5 — Additional file 5: Table S5. Number of reported short variants in the two panels according to the sample type. [file 12920_2022_1230_MOESM5_ESM.docx]

**Table S5.** Number of reported short variants in the two panels according to the sample type

| Sample type | TO panel | TN panel | Both panels | Concordance rate |
| --- | --- | --- | --- | --- |
| FF | 15 | 2 | 20 | 54.1% |
| FFPE-H | 28 | 11 | 10 | 20.4% |
| FFPE-L | 56 | 18 | 21 | 22.1% |

TO: Tumor-only, TN: tumor–normal.
