# Supplementary material for: Inter-assay variability of next-generation sequencing-based gene panels
Source: BMC Med Genomics. 2022 Apr 15;15:86. doi: 10.1186/s12920-022-01230-y (PMC9013031; doi:10.1186/s12920-022-01230-y)
Supplement: Supplementary file 6 — Additional file 6: Table S6. Analysis of discordant variants that were detected only in one panel. [file 12920_2022_1230_MOESM6_ESM.docx]

**Table S6.** **Analysis of discordant variants that were detected only in one panel**

| **Only in the**  **TO panel** | **Found in control samples of the TN panel** | **Filtered after**  **variant calling** | **Not detected by**  **variant caller** |
| --- | --- | --- | --- |
| FF | 15 | 0 | 0 |
| FFPE_H | 15 | 1 | 11 |
| FFPE_L | 2 | 0 | 55 |

| **Only in the**  **TN panel** | **Not in the TO panel’s**  **target genes** | **Filtered after**  **variant calling** | **Not detected by**  **variant caller** |
| --- | --- | --- | --- |
| FF | 0 | 2 | 0 |
| FFPE_H | 2 | 3 | 6 |
| FFPE_L | 0 | 3 | 15 |

N/A: not available
