# Supplementary material for: Inter-assay variability of next-generation sequencing-based gene panels
Source: BMC Med Genomics. 2022 Apr 15;15:86. doi: 10.1186/s12920-022-01230-y (PMC9013031; doi:10.1186/s12920-022-01230-y)
Supplement: Supplementary file 7 — Additional file 7: Table S7. Number of point mutations in the variants reported in the tumor-only and tumor–normal panels. [file 12920_2022_1230_MOESM7_ESM.docx]

**Table S7.** Number of point mutations in the variants reported in the tumor-only and tumor–normal panels

| Sample type | DNA alteration | TO panel | Both panels | TN panel |
| --- | --- | --- | --- | --- |
| FF | C>A | 2 | 4 | 1 |
|  | C>G | 3 | 2 | 0 |
|  | C>T | 6 | 8 | 1 |
|  | T>A | 0 | 1 | 0 |
|  | T>C | 4 | 2 | 0 |
|  | T>G | 0 | 0 | 0 |
| FFPE-H | C>A | 4 | 0 | 1 |
|  | C>G | 2 | 2 | 0 |
|  | C>T | 12 | 7 | 8 |
|  | T>A | 3 | 0 | 0 |
|  | T>C | 2 | 1 | 0 |
|  | T>G | 2 | 0 | 0 |
| FFPE-L | C>A | 8 | 3 | 0 |
|  | C>G | 0 | 2 | 0 |
|  | C>T | 43 | 8 | 15 |
|  | T>A | 3 | 0 | 0 |
|  | T>C | 3 | 5 | 2 |
|  | T>G | 0 | 1 | 0 |

TO: Tumor-only, TN: tumor–normal.
