# Supplementary material for: Inter-assay variability of next-generation sequencing-based gene panels
Source: BMC Med Genomics. 2022 Apr 15;15:86. doi: 10.1186/s12920-022-01230-y (PMC9013031; doi:10.1186/s12920-022-01230-y)
Supplement: Supplementary file 8 — Additional file 8: Table S8. Tumor cellularity in samples used for the first and second tumor–normal panel assays. [file 12920_2022_1230_MOESM8_ESM.docx]

**Table S8.** Tumor cellularity in samples used for the first and second tumor–normal panel assays

| Group | Sample No. | First sample | Second sample |
| --- | --- | --- | --- |
| FFPE-H | 11 | 30% | <10% |
|  | 12 | 20% | 30% |
|  | 13 | 10% | 10% |
|  | 14 | 50% | 50% |
|  | 15 | 20% | 20% |
|  | 16 | 40% | 40% |
|  | 17 | 60% | 60% |
|  | 18 | 10% | 10% |
|  | 19 | 10% | 10% |
|  | 20 | 70% | 70% |
| FFPE-L | 21 | 30% | 30% |
|  | 22 | 20% | 20% |
|  | 23 | 40% | 40% |
|  | 24 | 50% | 50% |
|  | 25 | <5% | <5% |
|  | 26 | 10% | 10% |
|  | 27 | 30% | 30% |
|  | 28 | 10% | 10% |
|  | 29 | 15% | <10% |
|  | 30 | 30% | 30% |
