# Supplementary material for: Inter-assay variability of next-generation sequencing-based gene panels
Source: BMC Med Genomics. 2022 Apr 15;15:86. doi: 10.1186/s12920-022-01230-y (PMC9013031; doi:10.1186/s12920-022-01230-y)
Supplement: Supplementary file 9 — Additional file 9: Table S9. Number of point mutations in the concordant and discordant variants reported in the two different tumor–normal panel assays. [file 12920_2022_1230_MOESM9_ESM.docx]

**Table S9.** Number of point mutations in the concordant and discordant variants reported in the two different tumor–normal panel assays

| Sample type | DNA alteration | Concordant variant | Discordant variant |
| --- | --- | --- | --- |
| FFPE-H | C>A | 1 | 1 |
|  | C>G | 1 | 1 |
|  | C>T | 10 | 9 |
|  | T>C | 0 | 2 |
|  | T>G | 0 | 0 |
| FFPE-L | C>A | 3 | 1 |
|  | C>G | 2 | 3 |
|  | C>T | 11 | 25 |
|  | T>C | 5 | 2 |
|  | T>G | 1 | 0 |
