# Supplementary figures and images for: Inter-assay variability of next-generation sequencing-based gene panels
Source: BMC Med Genomics. 2022 Apr 15;15:86. doi: 10.1186/s12920-022-01230-y (PMC9013031; doi:10.1186/s12920-022-01230-y)

**Figure S1**

**A**

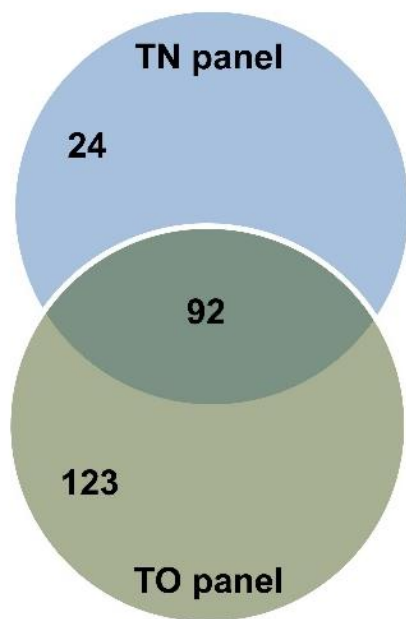

**B**

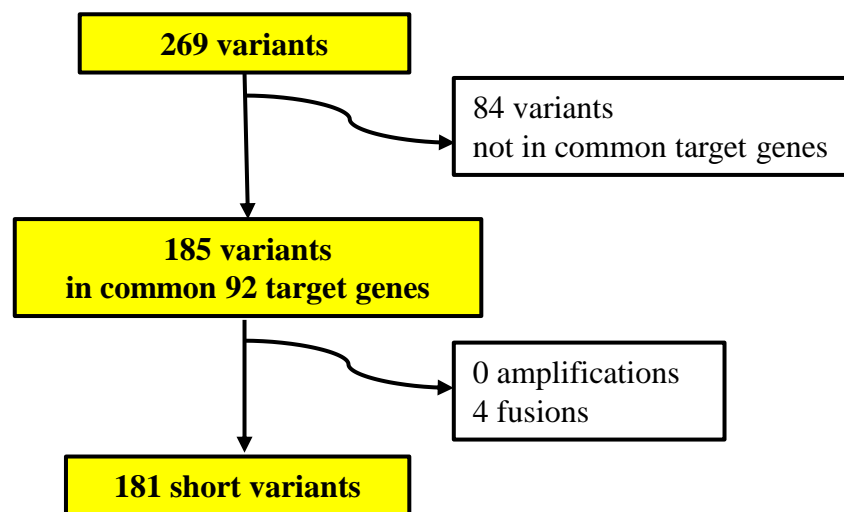

Supplement: Supplementary file 10 — Additional file 10: Figure S1. The working flow of comparative analysis of tumor variants. (A) The overlapping and different genes between the tumor-only (TO) panel and tumor–normal (TN) panel. (B) Type and number of alterations that were reported and filtered during the comparative analysis. [file 12920_2022_1230_MOESM10_ESM.pdf]

Figure S2

A

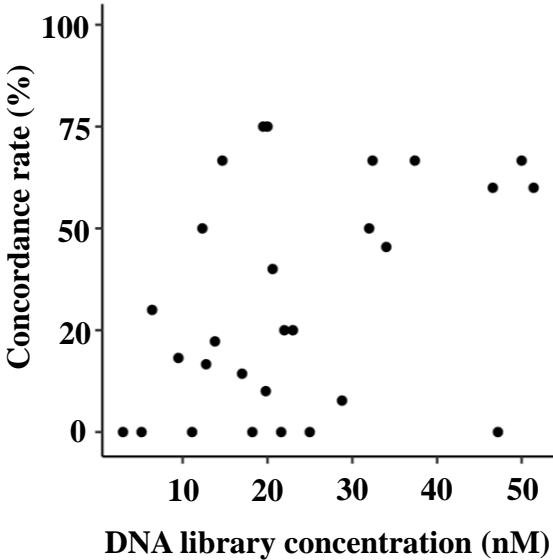

B

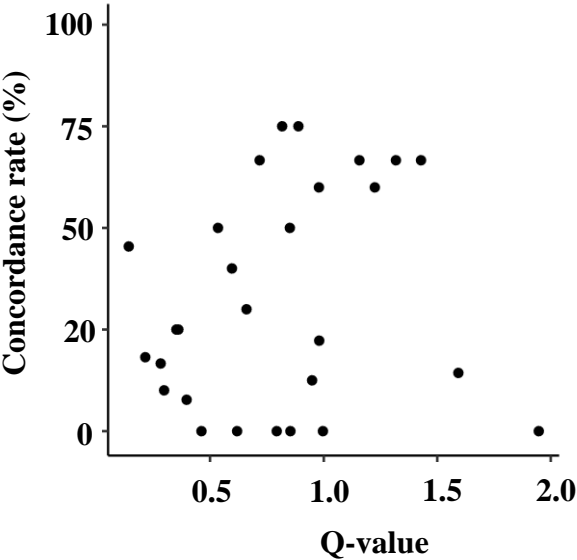

Supplement: Supplementary file 11 — Additional file 11: Figure S2. DNA quality and concordance rate between the two panels. (A) Distribution of reported variants in terms of concordance rate between the two panels (vertical axis) and DNA library concentration (horizontal axis). (B) Distribution of reported variants in terms of concordance rate between the two panels (vertical axis) and Q-value (horizontal axis). [file 12920_2022_1230_MOESM11_ESM.pdf]

**Figure S3**

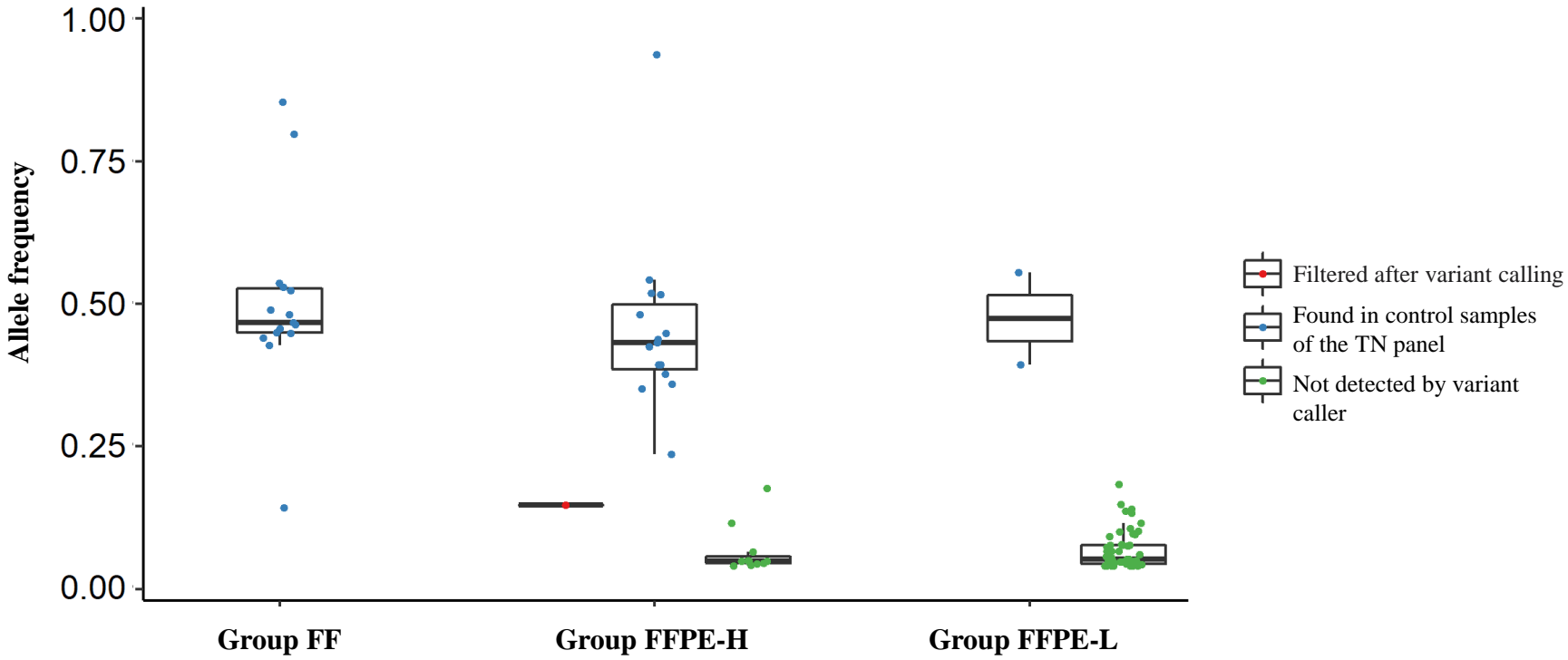

Supplement: Supplementary file 12 — Additional file 12: Figure S3. Analysis of discordant variants that were reported only in the tumor-only panel. A total of 99 variants detected only in the tumor-only (TO) panel were classified into five categories according to possible causes of discordance and mapped with allele frequency in Group FF, FFPE-H, and FFPE-L. [file 12920_2022_1230_MOESM12_ESM.pdf]

**Figure S4**

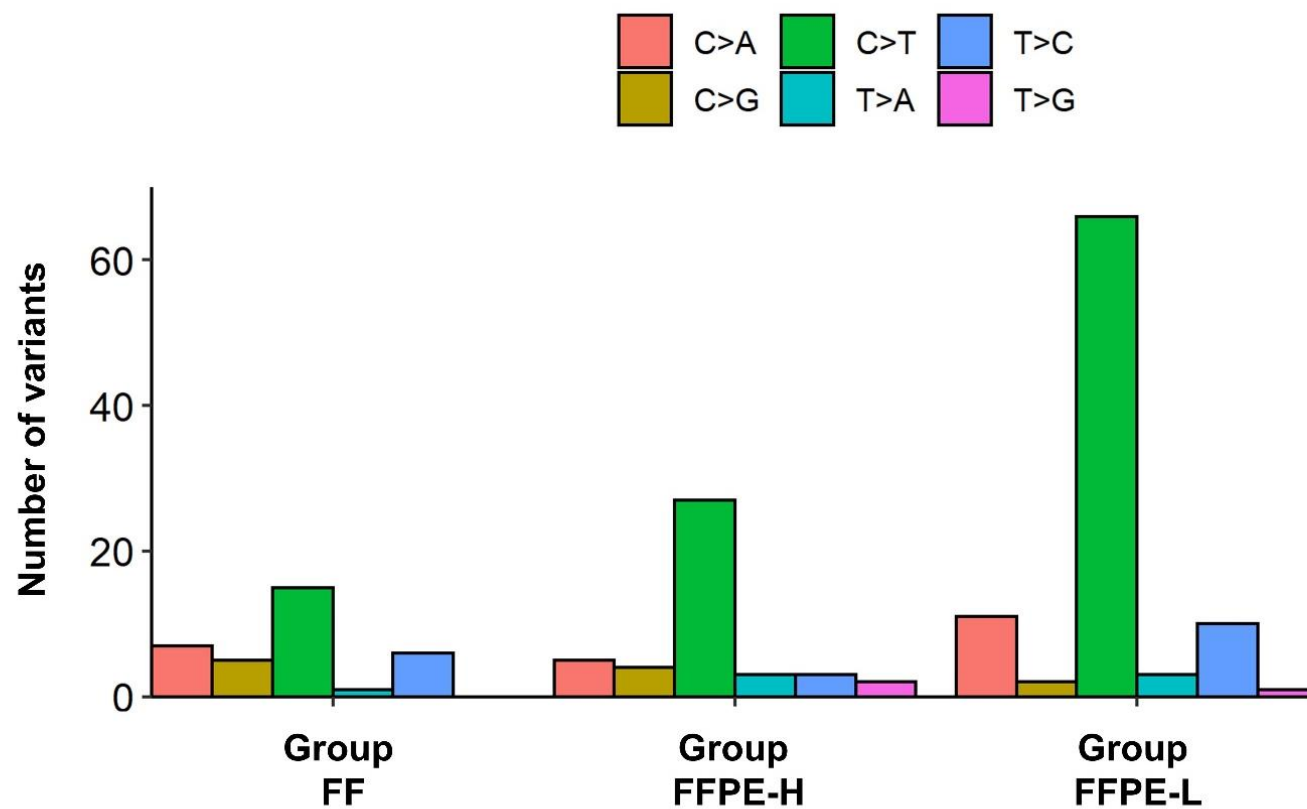

Supplement: Supplementary file 13 — Additional file 13: Figure S4. Incidence of point mutations in short variants reported only in the tumor-only panel. The incidence of C>T/G>A mutations in samples belonging to Group FF, FFPE-H and FFPE-L. [file 12920_2022_1230_MOESM13_ESM.pdf]
